# Investigation of Nano-Scale Segregation in Nanostructured Ferritic Alloy 14YWT after Heavy Ion Irradiation

**DOI:** 10.3390/ma15207257

**Published:** 2022-10-17

**Authors:** Junfeng Cai, Wentuo Han, Farong Wan, Jianchao He

**Affiliations:** 1 Institute of Special Environments Physical Sciences, Harbin Institute of Technology, Nanshan District, Shenzhen 518055, China; 2 School of Materials Science and Engineering, University of Science and Technology Beijing, Beijing 100083, China

**Keywords:** atom probe tomography, oxide-dispersion-strengthened (ODS) steels, segregation, nano oxide particles

## Abstract

Oxide-dispersion-strengthened (ODS) steels, which contain nano-scale Y-Ti-O particles, are being considered for high-temperature radiation environments of nuclear reactors. It is important to accurately characterize the structure of grain boundaries and understand the behavior of segregation at grain boundaries in ODS steels during irradiation. The effect of heavy ion irradiation at 700 °C on Nanostructured Ferritic Alloy 14YWT was investigated using Atom Probe Tomography. Enrichment of Cr occurs at the grain boundaries as well as at nano oxide particle surfaces in the unirradiated sample. The enrichment of Ti and Y at a grain boundary corresponds with Y-Ti-O nano oxide particles with larger size compared to those in the grain, and the Cr enrichment is particularly accentuated at these larger nano oxide particles. The segregation of W occurs at the grain boundaries that are without nano oxide particles. O is segregated at grain boundaries without oxide particles after irradiation. The segregation behavior of Cr, W, Ti, and Y at the grain boundary in the irradiated samples is similar to that in the unirradiated sample. The nano oxide particles embedded in the grain boundary are a primary reason for the increase in Cr segregation at the grain boundary.

## 1. Introduction

Oxide-dispersion-strengthened (ODS) steels are being considered as one of candidate materials for fuel cladding for Generation IV fission reactors and for blanket structural components for fusion reactors [1,2]. ODS steels are normally produced by mechanical alloying (MA) of initial powders and consolidated by hot isostatic pressing (HIP) or hot extrusion [3,4]. ODS steels have superior creep rupture strength due to the presence of nano-scale oxide particles distributed uniformly in the ferritic matrix. The nano oxide particles in the ODS steel are presumed to play an important role in radiation resistance by acting as sites for helium and the recombination of point defects formed during irradiation [5].

In ODS steels, oxide nano oxide particles are composed predominantly of Y, Ti, and O in the size range of about 2–50 nm [6,7]. The nano oxide particles are distributed mostly in the grains, but some of them have also been detected at the grain boundaries by TEM [8]. Many previous studies on ODS steels have focussed on the stability of nano oxide particles during irradiation by atom probe tomography (APT) and transmission electron microscope TEM [9,10,11,12,13]. A few investigations of segregation in ODS steels [14,15,16] have been carried out, particularly in regard to understanding the segregation at the grain boundaries when some nano oxide particles are distributed in the grain boundary [17].

The nano oxide particles embedded in the grain boundary may affect the spatial distribution of solute atoms along the grain boundaries. Given the important role played by nano oxide particles in the ODS steel, it is vital that this feature of segregation in the ODS steel is clearly understood. In this paper, one of the grain boundaries in the irradiated and unirradiated samples was selected for detailed analysis. The main purpose of this paper is to deeply explore the effect of oxide particles on element segregation. In the past, research on radiation segregation of ODS steel has mainly focused on the whole grain boundary and ignored the effect of oxide particles on the radiation segregation when the grain boundary aggregates, so this paper does not select more grain boundaries for research. This study is an initial step towards a series of more systematic investigations of radiation-induced segregation (RIS) in ODS steels.

## 2. Materials and Methods

**Figure 1:**
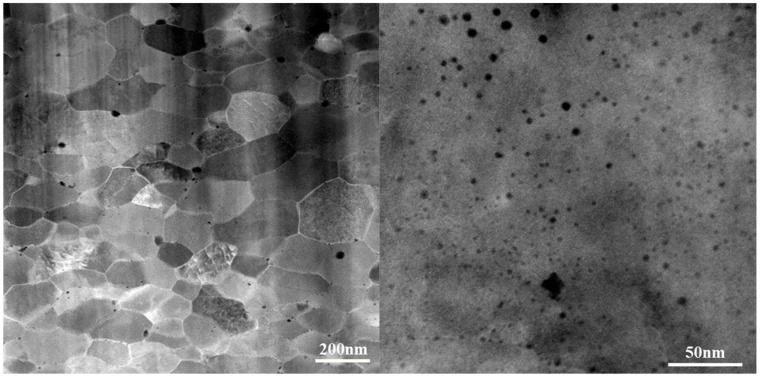
HAADF STEM (high-angle annular dark-field scanning transmission electron microscopy) images showing the microstructure of the 14YWT Alloy.

The nanostructured ferritic alloy 14YWT with the single phase of body-centered cubic structure (BCC) (heat SM10, nominal composition: Fe-14 wt% Cr-3 wt% W-0.4 wt% Ti-0.25 wt% Y_2_O_3_ and C is less than 0.1%) was provided by Oak Ridge National Laboratory (ORNL) and was prepared by mechanical alloying of a mixture of pre-alloyed ferritic steel and Y_2_O_3_ powders extruded at 850 °C, annealed at 1000 °C for 1 h, and finally hot-rolled at 850 °C for a 40% reduction. The details of sample fabrication and analysis of unirradiated samples are discussed elsewhere [9,18,19]. Figure 1 shows the grain structure (~200–400 nm) of the as-received 14YWT alloy with a high number density of nano oxide particles from 2 to 50 nm in size with some nano oxide particles distributed along the grain boundaries.

Ion irradiations were performed at Pacific Northwest National Laboratory (PNNL) by an NEC Pelletron tandem accelerator using 5 MeV Ni^2+^ ions at 700 °C, with an ion fluence of 1.49 × 10^21^ m^−2^. The Ni ion flux was 1.04 × 10^17^ m^−2^/s^−1^, corresponding to a damage rate of 1.39 × 10^−2^ dpa/s at the damage peak (~1600 nm), up to 200 dpa. A filament heater was mounted behind the specimen stage to obtain the desired irradiation temperature that was controlled by an electron beam for heating and by a copper alloy heat sink cooled by liquid nitrogen. This approach allowed for bracketing the sample temperature within an error range of ±10 °C.

**Figure 2:**
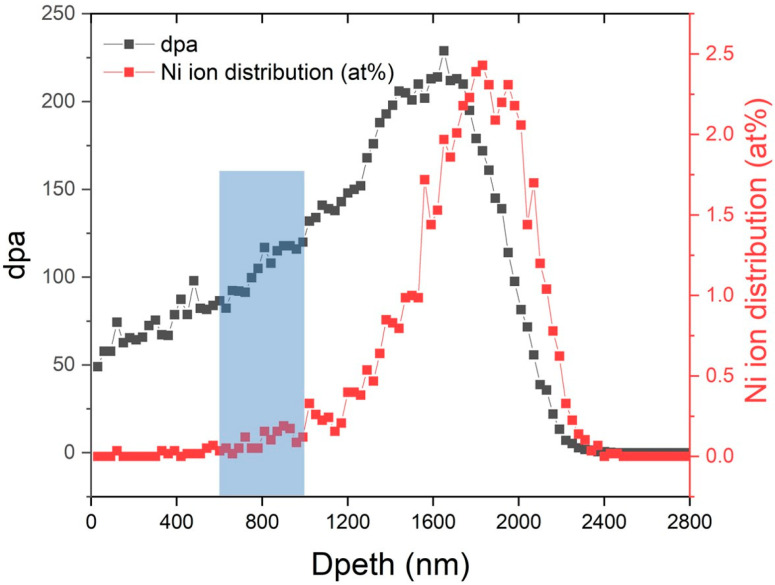
SRIM predictions of dpa damage and Ni concentration (at%) as a function of depth below the surface of the steel.

The depth profile of the damage for 5MeV Ni^2+^ ions irradiated of 14YWT alloy was evaluated using SRIM 2013 Pro (Stopping and Range of Ions in Matter) with 5 MeV Ni in the “Quick” Kinchin and Pease mode [20], and the results are shown in Figure 2. The specimens were sectioned at depths of 500 nm to 700 nm for APT analysis.

Samples for APT analysis were prepared using an FEI Quanta 3D FEG (Hillsboro, OR, USA). focused ion beam (FIB) instrument and analyzed in a voltage-pulse mode with a Cameca Instruments local electrode atom probe (LEAP 4000X HR, Fitchburg, WI, USA). The sample temperature for APT analysis was 55 K, and a pulse repetition rate of 200 kHz and a pulse fraction of 0.2 were used. The Integrated Visualization and Analysis Software (IVAS) Version 3.6.6 were used to perform three-dimensional (3-D) reconstruction and compositional analysis of nano oxide particles by isoconcentration surfaces. Three-dimensional isoconcentration surfaces were determined using Imago IVAS software 3.66 with a voxel size of 1 nm^3^ and using a smoothing algorithm having Gaussian form with an overall width of 2 nm. The composition profiles across the isoconcentration interfaces were constructed using the proximity histogram (proxigram) method [21]. Composition profiles across a grain boundary were produced by performing a composition analysis along a cylinder that crosses the grain boundary. The cylinder was 5 nm in diameter with a bin size of 0.5 nm along the cylinder to evaluate the composition. The average radius and number density of Ti-Y-O nanoclusters were determined using the maximum separation method, which can distinguish solute elements in the nanoclusters from the atoms constituting matrix elements. A maximum separation distance of 0.66 nm and a minimum size limit of 8 atoms were used in this study. Ions Ti, Y, YO, and TiO were used to create cluster analysis and to identify nanoclusters.

## 3. Results and Discussion

### 3.1. Characterization of the Grain Boundary in the ODS Steels Using APT

**Figure 3:**
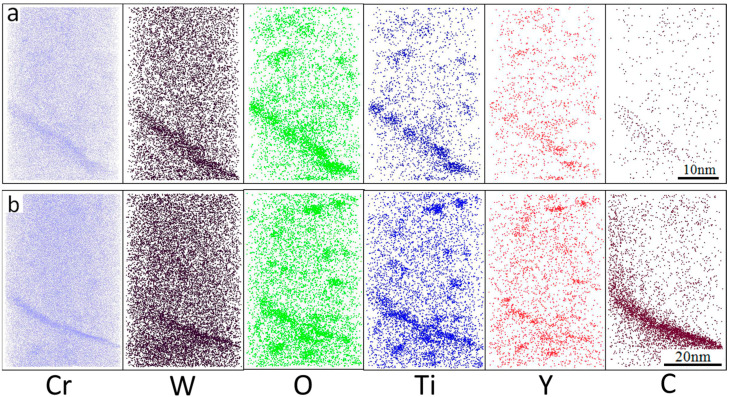
APT atom maps of elements in the 14YWT alloy (**a**) before heavy ion irradiation and (**b**) after heavy ion irradiation to 100 dpa at 700 °C.

**Figure 4:**
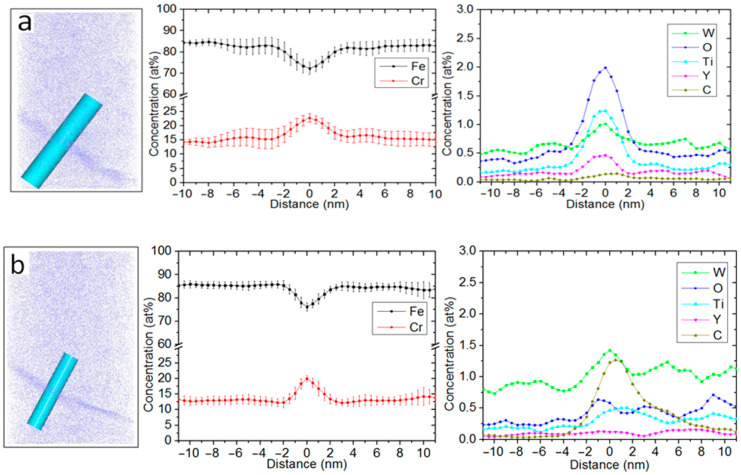
Concentration profiles across a grain boundary in 14YWT alloy (**a**) before heavy ion irradiation and (**b**) after heavy ion irradiation to 100 dpa at 700 °C.

Three-dimensional atom maps of the unirradiated 14YWT APT sample and a sample irradiated with 5MeV Ni^2+^ at 700 °C up to 100 dpa reconstructed by IVAS are shown in Figure 3a,b. The average Guinier radii of the unirradiated sample and of samples irradiated at 700 °C were 1.08 ± 0.52 and 1.08 ± 0.61 nm, respectively, and corresponding number densities were 12 × 10^23^ and 6.0 × 10^23^ n/m^−3^. The non-uniform distributions of Cr, W, O, Ti, Y, and C atoms are evident in these individual atom maps and are indicative of enrichment in both samples. Cr, W, O, Ti, and Y enriched in the grain boundaries by 8.09 at%, 0.44 at%, 1.62 at%, 1.04 at%, and 0.37 at%, respectively, and Fe was depleted by −11.58 at%, compared to the unirradiated sample, as shown in Figure 4a,b, as summarized in Table 1. Other work has noted significant enrichment of Cr, W, Ti, Y, O, and C around the core of a dislocation in the 12YWT alloy using APT [22].

**Figure 5:**
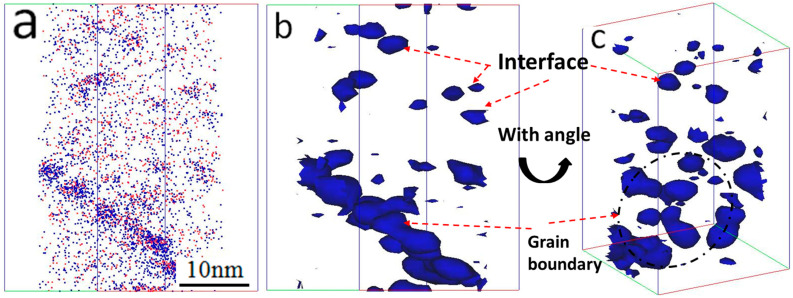
(**a**) Ti + Y atom map and (**b**,**c**) Ti interfaces defined by isoconcentration surfaces of 1.5 at% Ti from two angles in unirradiated 14YWT alloy.

Ti atomic isoconcentration surfaces were used to define nano oxide particles, since the nano oxide particles are composed of Y, Ti, and O in the 14YWT alloy. A high number density of nano oxide particles in the 14YWT samples characterized by Ti atomic isoconcentration surfaces are shown in Figure 5a,b. To establish the geometrical form of grain boundaries and identify that the segregation happened at grain boundaries or dislocations, isoconcentration surfaces corresponding to 1.5 at% of Ti, viewed from two directions, were constructed and are shown in Figure 5b,c. The geometry of the grain boundary regions can be seen to be a plate-like area, while the dislocation is a line-like geometry in the 3-D maps [21]. A plate-like area is observed from the segregation after rotating through an angle, indicating that the segregation area is a grain boundary. The nano oxide particles with larger sizes are embedded in the grain boundary than those distributed in the grain, as shown in Figure 5.

**Figure 6:**
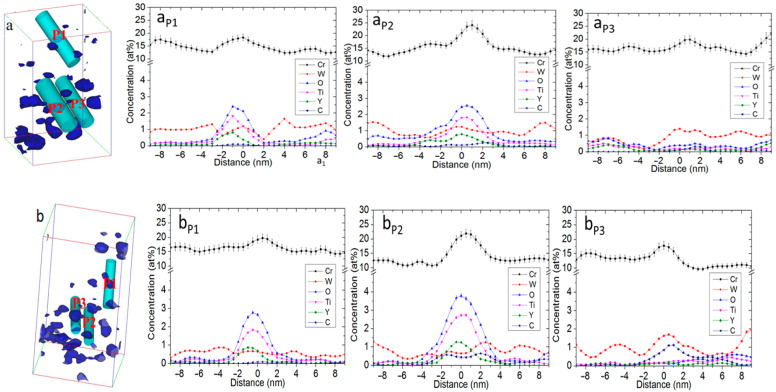
The three selected different positions in the 14YWT ((**aP_1_**,**bP_1_**) is in the matrix containing a nano oxide particle; (**aP_2_**,**bP_2_**) crosses the grain boundary with a nano oxide particle; (**aP_3_**,**bP_3_**) crosses the grain boundary without the nano oxide particle; (**aP_1_**) is in the sample without heavy ion irradiation; (**bP_1_**) is in the sample with heavy ion irradiation) and their elements’ distributions (at%) along the volume in the 14YWT alloy (**a**) before heavy ion irradiation and (**b**) after heavy ion irradiation up to 100 dpa at 700 °C.

Three different positions in the APT reconstructed 3-D maps of unirradiated and irradiated 14YWT alloy were selected to investigate the effect of nano oxide particles distributed on the segregation at grain boundary in the ODS steels as shown in Figure 6a,b. P_1_ is in the matrix grain containing a nano oxide particles; P_2_ crosses the grain boundary with nano oxide particles; P_3_ crosses the grain boundary without the nano oxide particles. The segregation of Ti and Y happened in the P_2_ position, not in the P_3_ position, indicating that the enrichment of Ti and Y at the grain boundary is in the form of Ti-Y-O nano oxide particles. However, the W enriched in the position P_3_ rather than P_2_, revealing that the segregation of W happened in the grain boundaries without nano oxide particles. Cr segregation is noted for all three positions, as shown Figure 6(aP_1_–aP_3_). Cr was segregated in the positions P_1_, P_2_, and P_3_ by 2.85%, 8.82%, and 5.18%, respectively, compared to the bulk, as shown in Table 1.

**Table 1:** Average measured composition of bulk, grain boundary, and particles in the position P_1_, P_2,_ and P_3_ referred to in Figure 6.

Chemical Composition (at%)							
	Fe	Cr	W	O	Ti	Y	C
Unirradiated							
Bulk	83.93 ± 1.35	14.71 ± 0.20	0.58	0.37	0.19	0.09	0.06
GB (Δ)	72.35 ± 2.93 (−11.58)	22.80 ± 1.45 (8.09)	1.02 ± 0.45 (0.44)	1.99 ± 1.12 (1.62)	1.23 ± 0.83 (1.04)	0.46 ± 0.33 (0.37)	0.14 ± 0.11 (0.08)
P1 (Δ)	75.40 ± 5.99 (−8.53)	17.56 ± 1.23 (2.85)	0.95 ± 0.01 (0.37)	2.40 ± 0.07 (2.03)	1.81 ± 0.04 (1.62)	0.82 ± 0.0 (0.73)	0.10 (0.04)
P2 (Δ)	69.21 ± 5.44 (−14.72)	23.53 ± 1.70 (8.82)	1.13 ± 0.02 (0.55)	2.56 ± 0.07 (2.19)	1.83 ± 0.04 (1.64)	0.70 ± 0.01 (0.61)	0.13 (0.07)
P3 (Δ)	77.41 ± 6.76 (−6.52)	19.89 ± 1.66 (5.18)	1.22 ± 0.03 (0.64)	0.42 ± 0.01 (0.05)	0.14 (-0.05)	0.16 (0.07)	0.22 (0.16)
After irradiation							
Bulk	83.36 ± 1.35	15.1 ± 0.23	0.58	0.39	0.13	0.07	0.06
GB (Δ)	76.13 ± 1.76 (−7.23)	20.01 ± 1.03 (4.91)	1.42 ± 0.54 (0.84)	0.58 ± 0.22 (0.19)	0.47 ± 0.15 (0.34)	0.13 ± 0.11 (0.06)	1.22 ± 0.39 (1.16)
P1 (Δ)	74.90 ± 5.80 (−8.46)	19.27 ± 1.36 (4.17)	0.64 ± 0.01 (0.06)	2.66 ± 0.08 (2.27)	1.74 ± 0.04 (1.61)	0.69 ± 0.01 (0.62)	0.09 (0.03)
P2 (Δ)	69.50 ± 5.02 (−13.86)	21.96 ± 1.42 (6.86)	0.67 ± 0.01 (0.09)	3.63 ± 0.10 (3.24)	2.73 ± 0.07 (2.6)	1.07 ± 0.02 (1.00)	0.43 (0.37)
P3 (Δ)	79.13 ± 6.78 (−4.23)	17.86 ± 1.44 (2.76)	1.63 ± 0.04 (1.05)	0.16 (-0.23)	0.19 (0.06)	0.16 (0.09)	0.86 (0.8)

### 3.2. The Relationship of Nano Oxide Particles and Cr Segregation in the ODS Steel

**Figure 7:**
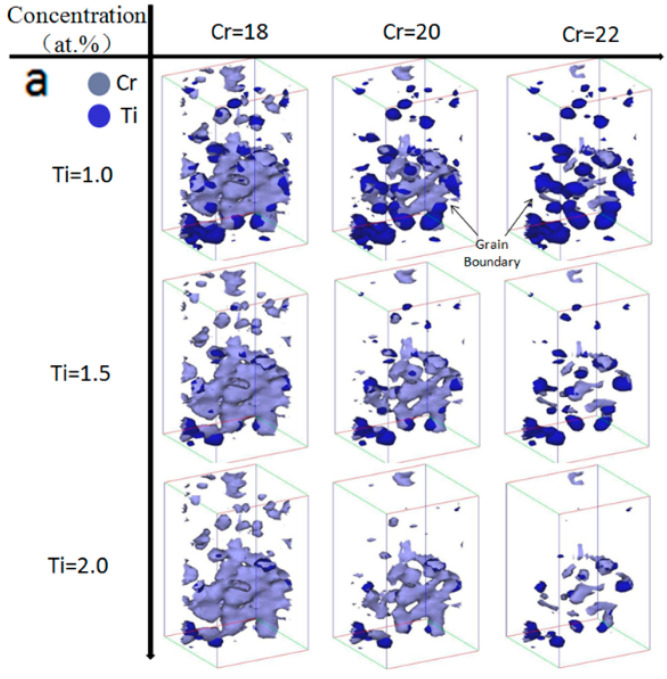

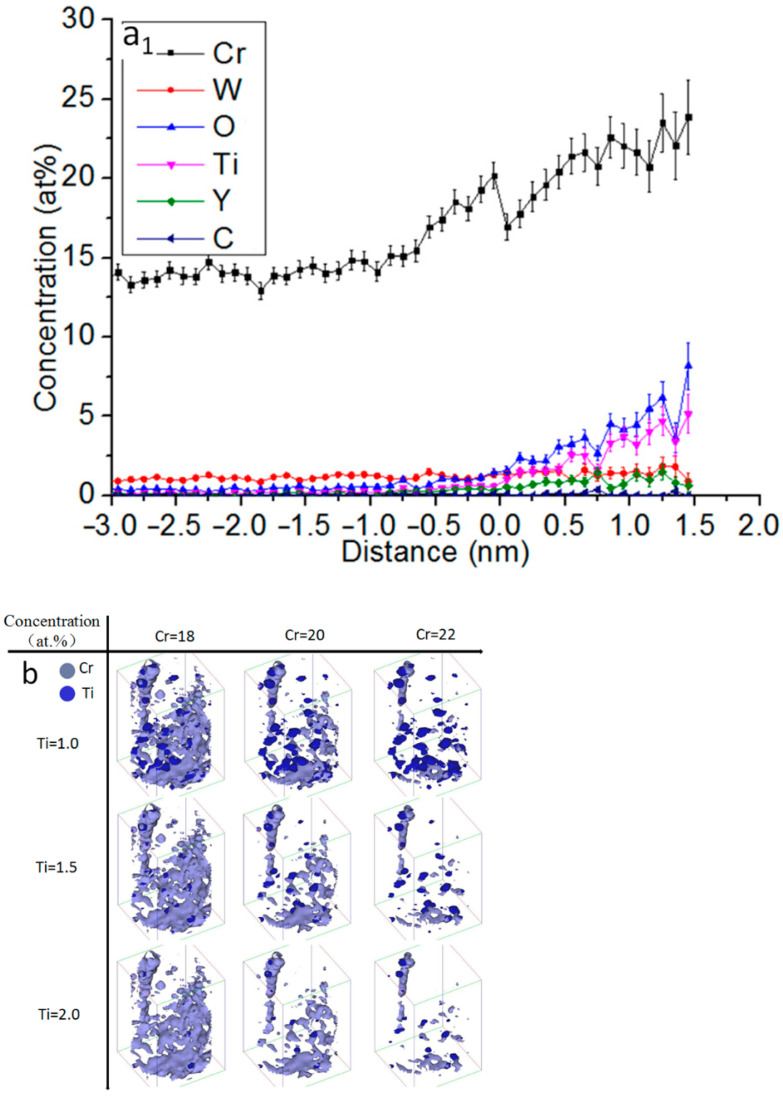

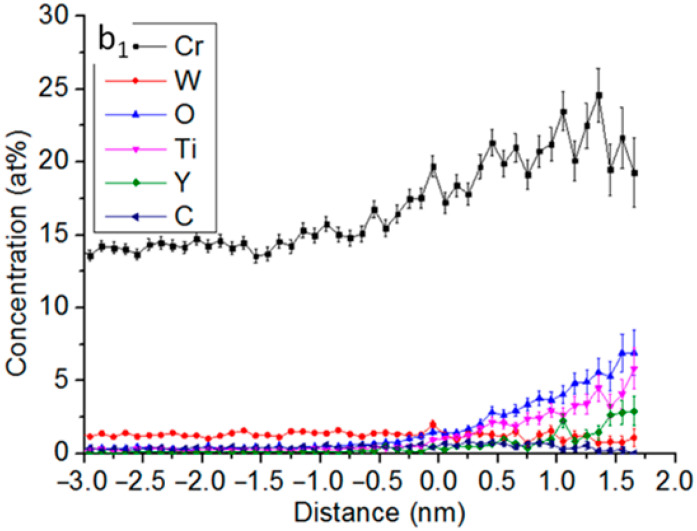
Ti interfaces and Cr interfaces defined by isoconcentration surfaces of 1.0 to 2.0 at% Ti and 18 to 22 at% Cr in 14YWT, and the corresponding proxigram showing variation in composition as a function of distance from an 18% Cr isoconcentration surface in 14YWT alloy (**a**) before heavy ion irradiation and (**b**) after heavy ion irradiation to 100 dpa at 700 °C. ((**a1**) is the corresponding proxigram of 18% Cr isoconcentration surface in 14YWT alloy without heavy ion irradiation, (**b1**) is the corresponding proxigram of 18% Cr isoconcentration surface in 14YWT alloy with heavy ion irradiation).

The concentration of Cr in position P_2_ is higher than it is in position P_3_, indicating that the segregation of Cr occurs around nano oxide particles. Figure 6a shows the relationship between the distribution of Cr and the nano oxide particles by observing together the change in the isoconcentration surfaces of Ti (the concentration of Ti is 1.0 to 2.0 at%) and Cr (the concentration of Cr is 1.5 to 2.5 at%). When the concentration of Ti is 2.0%, the isoconcentration surface of Ti disappears in the grain, and it still appears in the grain boundary. The results indicate that the nano oxide particles distributed in the grain boundary contained a higher concentration of Y, Ti, and O solute atoms. Similar results were also found for Cr. With the increase in Cr concentration, the zone enclosed with Cr isoconcentration surface shrank around the nano oxide particles. The concentration Cr at the interface between nano oxide particles and matrix was higher than at other positions. When the concentration of Cr was 22%, the isoconcentration surface of Cr in the grain almost disappeared, but it still appeared in the grain boundary, revealing that it is preferred for Cr to segregate around the nano oxide particles in the grain boundary. These results can be inferred by the method of proxigram, which is described using the concentration of elements across the nano oxide particles as shown in Figure 7a. The concentration of Cr obviously increased from the matrix to the nano oxide particles and then decreased near the core of nano oxide particles. This agrees with compositions of nano oxide particles measured by the proxigram method in the Fe-14Cr alloy and MA957 alloys [23,24], particularly in terms of Y, Ti, and Cr content. Other studies have noted the formation of a Cr-rich shell of the nano oxide particles [25,26,27]. The segregation of Cr in the position P_2_ is higher than it is in P_1_, and the full width at the half maximum of Ti, Y, and O of the nano oxide particles in P_2_ is wider than that in P_1_, as shown in Figure 6a, indicating that the grain boundaries contained larger nano oxide particles. One reason for the nano oxide particles with higher Cr being in the grain boundary is that the size of nano oxide particles is larger than that in the grain. The nano oxide particles in grain boundary with significantly larger (5–10 nm) size than those in the interior of grain were also observed in the MA957 [20]. Marquis noted that the segregation of Cr is enhanced in some nano oxide particles, and the distinction between core and shell becomes clearer as the particle size increases, supporting the contention that Cr segregated to the ultrafine nano oxide particles and the larger-sized nano oxide particles may have a thicker Cr shell, which makes higher Cr segregation around nano oxide particles [28].

### 3.3. Irradiation Induced Segregation in the 14YWT

In the irradiated samples at 700 °C with a damage level up to 100 dpa, Y, Ti, and O nano oxide particles with high number density still existed in the grain and grain boundaries. The segregation of Cr, W, O, Ti, Y, and C at the grain boundary is observed in Figure 3b. Figure 4b shows representative composition profiles across the grain boundary. Cr was segregated by 4.91 at%, O by 0.19 at%, Ti by 0.34 at%, Y by 0.06 at%, and W by 0.84 at%, and Fe was depleted by 2.7 at% at grain boundary in the irradiated samples. The full width at the half maximum of the profiles was about 1 nm. The relationship between the distribution of Cr and nano oxide particles in the irradiated samples shows that Cr with a higher concentration segregated at the interface of nano oxide particles with a Cr-rich shell, which is similar to unirradiated samples, as shown in Figure 7(b,b1). Some nano oxide particles were still distributed at the grain boundary in the irradiated samples, which made the distribution of Cr non-uniformed in the grain boundary. Cr was enriched in P_1_, P_2,_ and P_3_ by 4.17%, 6.86%, and 2.76%, respectively, compared to the bulk, as shown in Table 1. The concentration of Cr in P_2_ is highest in the three positions and the segregation of W happened in the grain boundary without nano oxide particles, as shown in Figure 6(bP_1_–bP_3_). Marquis et al. [15] observed Cr-enrichment at grain boundaries in the unirradiated Fe-12Cr wt% ODS. After irradiation at 500 °C with the 2 MeV Fe ion, the results revealed a complex distribution of Cr enrichment and depletion at grain boundaries of varying character. Hu et al. [16] performed irradiation at 350 °C with heavy ions on 12Cr-ODS steel. The results showed that Cr segregated to the grain boundary with C within a width of less than 8 nm but is slightly depleted in the vicinity of the grain boundary (1–2 nm) to yield a “W-shaped” profile. Was et al. [29] concluded that radiation-induced segregation is dependent on the alloy composition and irradiation temperature. Cr enrichment at grain boundaries occurred in the alloy with lower Cr (T91) and at lower temperature (400 °C), and depletion occurred in higher Cr alloys (HT9 and HCM12A) and at higher irradiation temperature (500 °C). There is no consensus in the literature as to whether Cr is segregated or depleted at the grain boundaries after irradiation, but the results from this study of Cr segregation in the unirradiated and sample irradiated at 700 °C up to 100 dpa reveal that the nano oxide particles embedded in the grain boundary are one of factors that increase the concentration of Cr at the grain boundary.

## 4. Conclusions

In summary, the structure of grain boundary of Nanostructured Ferritic Alloy 14YWT has been characterized using Atom Probe Tomography. The results showed that segregation of Cr happened at the grain boundaries as well as at the nano oxide particles. The grain boundaries contained larger sizes of nano oxide particles and the Cr segregation was particularly accentuated at these larger nano oxide particles. The enrichment of Ti and Y at the grain boundary is in the form of Ti-Y-O nano oxide particles, and the segregation of W happened in the grain boundary without nano oxide particles. After irradiation at 700 °C with a damage level up to 100 dpa, oxide particles always appeared within grains and grain boundaries with no significant change in size or density. In both irradiated and unirradiated samples, the oxide particles in the grain boundaries were larger than those in the grains. Oxygen was segregated at grain boundaries without oxide particles after irradiation. The segregation of Cr, W, O, Ti, and Y showed similar behavior to the unirradiated 14YWT alloy. The size of oxide particles had a certain influence on Cr segregation. With the increase in oxide particles, the segregation of Cr on oxide particles is more obvious. The nano oxide particles embedded in the grain boundary, which is one of factors of the increase in Cr concentration at the grain boundaries.
